# *trans*-Aqua­(4,7-diaza­decane-1,10-diamine-κ^4^ *N*)fluoridochromium(III) bis­(perchlorate) monohydrate

**DOI:** 10.1107/S1600536808026081

**Published:** 2008-08-20

**Authors:** Jong-Ha Choi, Uk Lee

**Affiliations:** a Department of Chemistry, Andong National University, Andong 760-749, Republic of Korea; b Department of Chemistry, Pukyong National University, 599-1 Daeyeon 3-dong Nam-gu, Busan 608-737, Republic of Korea

## Abstract

In the title compound, [CrF(C_8_H_20_N_4_)(H_2_O)](ClO_4_)_2_·H_2_O, the Cr atom is in a slightly distorted octa­hedral environment, coordinated by four N atoms of the 4,7-diaza­decane-1,10-diamine ligand, one water mol­ecule and an F atom *trans* to water. The five-membered chelate ring is in a *gauche* form, while the two six-membered chelate rings are in chair conformations. The crystal structure is stabilized by several hydrogen bonds.

## Related literature

For the synthesis, see: Glerup *et al.* (1970 ▶). For related structures, see: Brencic *et al.* (1985 ▶); Choi *et al.* (1995 ▶, 2004 ▶, 2006 ▶, 2008 ▶). For other related literature, see: Choi & Hoggard (1992 ▶); Poon & Pun (1980 ▶); Stearns & Armstrong (1992 ▶).
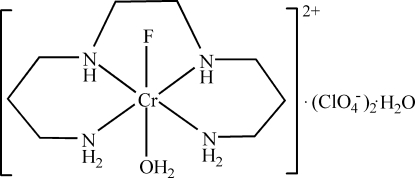


## Experimental

### 

#### Crystal data

[CrF(C_8_H_20_N_4_)(H_2_O)](ClO_4_)_2_·H_2_O
*M* *_r_* = 480.23
Monoclinic, 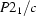

*a* = 9.950 (1) Å
*b* = 16.893 (2) Å
*c* = 12.008 (1) Å
β = 108.65 (1)°
*V* = 1912.4 (4) Å^3^
*Z* = 4
Mo *K*α radiation
μ = 0.94 mm^−1^
*T* = 298 (2) K
0.43 × 0.30 × 0.25 mm

#### Data collection

Stoe Stadi-4 diffractometer
Absorption correction: numerical (*X-SHAPE*; Stoe, 1996 ▶) *T*_min_ = 0.686, *T*_max_ = 0.889
4347 measured reflections
4347 independent reflections
3245 reflections with *I* > 2σ(*I*)
3 standard reflections frequency: 60 min intensity decay: 3.1%

#### Refinement

*R*[*F* ^2^ > 2σ(*F* ^2^)] = 0.076
*wR*(*F* ^2^) = 0.228
*S* = 1.09
4347 reflections
243 parameters
3 restraints
H atoms treated by a mixture of independent and constrained refinement
Δρ_max_ = 1.07 e Å^−3^
Δρ_min_ = −0.60 e Å^−3^

### 

Data collection: *STADI4* (Stoe & Cie, 1996 ▶); cell refinement: *STADI4*; data reduction: *X-RED* (Stoe & Cie, 1996 ▶); program(s) used to solve structure: *SHELXS97* (Sheldrick, 2008 ▶); program(s) used to refine structure: *SHELXL97* (Sheldrick, 2008 ▶); molecular graphics: *ORTEP-3* (Farrugia, 1997 ▶) and *DIAMOND* (Brandenburg, 1998 ▶); software used to prepare material for publication: *SHELXL97*.

## Supplementary Material

Crystal structure: contains datablocks global, I. DOI: 10.1107/S1600536808026081/cf2211sup1.cif

Structure factors: contains datablocks I. DOI: 10.1107/S1600536808026081/cf2211Isup2.hkl

Additional supplementary materials:  crystallographic information; 3D view; checkCIF report

## Figures and Tables

**Table 1:** Hydrogen-bond geometry (Å, °)

*D*—H⋯*A*	*D*—H	H⋯*A*	*D*⋯*A*	*D*—H⋯*A*
O1*W*—H1*OA*⋯F^i^	0.86 (6)	1.72 (6)	2.564 (5)	168 (8)
O1*W*—H1*OB*⋯O2*W*	0.74 (6)	1.92 (6)	2.617 (6)	158 (7)
N1—H1*AN*⋯O2^ii^	0.91	2.42	3.332 (11)	177
N2—H1*N*2⋯O7^iii^	0.91	2.45	3.154 (10)	134
N3—H3*BN*⋯O5	0.90	2.36	3.242 (10)	167
N4—H4*BN*⋯O5	0.90	2.43	3.062 (10)	127
C1—H1*B*⋯O4^iv^	0.97	2.53	3.141 (13)	121

Symmetry codes: (i) 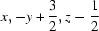; (ii) 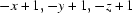; (iii) 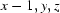; (iv) 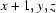.

**Fig. 1.**
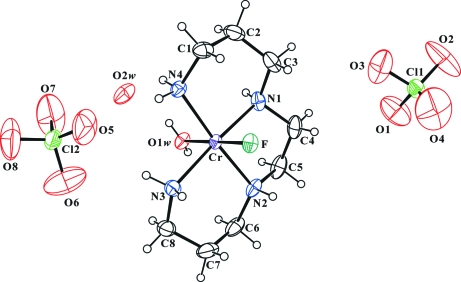
Molecular structure (30% probability ellipsoids) of the title compound.

**Fig. 2.**
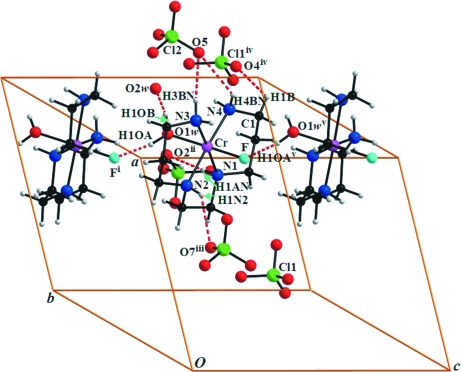
Hydrogen-bond interactions (dashed lines) in the title compound. [Symmetry codes: (i) x, -y+3/2, z-1/2; (ii) -x+1, -y+1, -z+1; (iii) x-1, y, z; (iv) x+1, y, z; (v) x, -y+3/2, z+1/2.]

## supplementary crystallographic information

### Comment

Acyclic flexible 4,7-diazadecane-1,10-diamine (3,2,3-tet) and its related tetradentate ligands provide a rich field of geometric and conformational isomers in octahedral transition metal complexes (Choi *et al.*, 2008). The electronic absorption and infrared spectra often can be used diagnostically to identify the geometric isomers of chromium(III) complexes complexes (Poon & Pun, 1980; Choi & Hoggard, 1992). However, it should be noted that the assignments based on spectroscopic investigations are not always conclusive (Stearns & Armstrong, 1992).  [Cr(3,2,3-tet)F(H_2_O)]X_2_ can adopt a diverse stereochemistry and configuration, but no structures have been reported. Thus we here report the crystal structure of the title complex (Fig. 1) in order to confirm the geometric configuration.

There are one fluorine atom and one water molecule coordinated to the chromium atom in a trans arrangement with an F—Cr—O1w bond angle of 179.5 (2)°. The rest of the coordination sites are occupied by four nitrogen atoms from 3,2,3-tet ligand in the equatorial plane. The mean Cr—N bond length of 2.079 (4) Å is normal, agreeing with literature values (Choi *et al.*,  1995; Choi *et al.*, 2004). The Cr—N1 and Cr—N2 bond lengths of 2.068 (5) and 2.070 (4) Å of secondary amines are slightly shorter than Cr—N3 and Cr—N4 distances of 2.082 (5) and 2.095 (4) Å of primary amines. The Cr—F distance of 1.881 (3) Å and Cr—O1W of 1.996 (4) Å are also compararble to the values of 1.870 (1) Å and 2.023 (2) Å found in trans-[Cr([15]aneN4)F_2_]ClO_4_ (Choi *et al.*,  2006) and trans-[Cr(NH_3_)_4_Cl(H_2_O)]Cl_2_ (Brencic *et al.*,  1985), respectively. The short Cr—F bond length suggests a strong bond.

The uncoordinated ClO_4_^-^ anions and one water molecule remain outside the coordination sphere. There is an extensive hydrogen bonding network (Table 2) between the oxygens of the ClO_4_^-^ anions, fluorine atom, water molecule, C—H and the N—H groups of the 3,2,3-tet ligand as shown in Figure 2. These hydrogen-bonded networks help to stabilize the crystal structure.

### Experimental

As starting material, trans-[Cr(3,2,3-tet)F_2_]ClO_4_ was prepared according to the literature (Glerup *et al.*,  1970). The complex trans-[Cr(3,2,3-tet)F_2_]ClO_4_ was dissolved in 0.2 M HClO_4_. The solution was heated at 333 K for 50 min and then a saturated solution of sodium perchlorate was added. Dark red crystals suitable for an X-ray structural determination were deposited over several days as the solution evaporated.

### Refinement

All H atoms were positioned geometrically and refined using a riding model, with C—H = 0.95 Å for aromatic H atoms and 0.98 Å for methyl H atoms, respectively, and with U_iso_(H) = 1.2U_eq_(C) for aromatic and U_iso_(H) = 1.5U_eq_(C) for methyl H atoms. H atoms of O1w were located in a difference Fourier map and refined with constraints.  Reasonable positions of H atoms for O2w could not obtained from a difference Fourier map.

### Crystal data

**Table:**

[CrF(C_8_H_20_N_4_)(H_2_O)](ClO_4_)_2_·H_2_O	*F*_000_ = 996
*M**_r_* = 480.23	*D*_x_ = 1.668 Mg m^−^^3^
Monoclinic, *P*2_1_/*c*	Mo *K*α radiation λ = 0.71069 Å
Hall symbol: -P 2ybc	Cell parameters from 32 reflections
*a* = 9.950 (1) Å	θ = 19.0–20.8º
*b* = 16.893 (2) Å	µ = 0.94 mm^−^^1^
*c* = 12.008 (1) Å	*T* = 298 (2) K
β = 108.65 (1)º	Block, dark red
*V* = 1912.4 (4) Å^3^	0.43 × 0.30 × 0.25 mm
*Z* = 4	

### Data collection

**Table:**

Stoe Stadi-4 diffractometer	*R*_int_ = 0.0000
Radiation source: fine-focus sealed tube	θ_max_ = 27.5º
Monochromator: graphite	θ_min_ = 2.2º
*T* = 298(2) K	*h* = −12→12
ω/2–θ scans	*k* = 0→21
Absorption correction: numerical(X-SHAPE; Stoe, 1996)	*l* = 0→15
*T*_min_ = 0.686, *T*_max_ = 0.889	3 standard reflections
4347 measured reflections	every 60 min
4347 independent reflections	intensity decay: 3.1%
3245 reflections with *I* > 2σ(*I*)	

### Refinement

**Table:**

Refinement on *F*^2^	Secondary atom site location: difference Fourier map
Least-squares matrix: full	Hydrogen site location: inferred from neighbouring sites
*R*[*F*^2^ > 2σ(*F*^2^)] = 0.076	H atoms treated by a mixture of independent and constrained refinement
*wR*(*F*^2^) = 0.228	*w* = 1/[σ^2^(*F*_o_^2^) + (0.1014*P*)^2^ + 6.2787*P*] where *P* = (*F*_o_^2^ + 2*F*_c_^2^)/3
*S* = 1.09	(Δ/σ)_max_ < 0.001
4347 reflections	Δρ_max_ = 1.07 e Å^−^^3^
243 parameters	Δρ_min_ = −0.60 e Å^−^^3^
3 restraints	Extinction correction: none
Primary atom site location: structure-invariant direct methods	

### Special details

**Table:**

Geometry. All esds (except the esd in the dihedral angle between two l.s. planes) are estimated using the full covariance matrix. The cell esds are taken into account individually in the estimation of esds in distances, angles and torsion angles; correlations between esds in cell parameters are only used when they are defined by crystal symmetry. An approximate (isotropic) treatment of cell esds is used for estimating esds involving l.s. planes.
Refinement. Refinement of F^2^ against ALL reflections. The weighted R-factor wR and goodness of fit S are based on F^2^, conventional R-factors R are based on F, with F set to zero for negative F^2^. The threshold expression of F^2^ > 2sigma(F^2^) is used only for calculating R-factors(gt) etc. and is not relevant to the choice of reflections for refinement. R-factors based on F^2^ are statistically about twice as large as those based on F, and R- factors based on ALL data will be even larger.

### Fractional atomic coordinates and isotropic or equivalent isotropic displacement parameters (Å^2^)

**Table:**

	*x*	*y*	*z*	*U*_iso_*/*U*_eq_	
Cr	0.77207 (8)	0.74161 (4)	0.61410 (6)	0.0294 (2)	
Cl1	0.25308 (17)	0.51481 (9)	0.64339 (13)	0.0538 (4)	
Cl2	1.24851 (16)	0.84876 (12)	0.62990 (16)	0.0643 (5)	
F	0.7140 (3)	0.74557 (18)	0.7485 (2)	0.0404 (7)	
N1	0.6809 (5)	0.6308 (3)	0.5748 (4)	0.0458 (11)	
H1AN	0.7106	0.6106	0.5164	0.055*	
N2	0.5732 (4)	0.7824 (3)	0.5160 (4)	0.0454 (11)	
H1N2	0.5272	0.7928	0.5688	0.055*	
N3	0.8507 (5)	0.8564 (3)	0.6485 (4)	0.0435 (10)	
H3AN	0.8338	0.8731	0.7139	0.052*	
H3BN	0.9455	0.8539	0.6654	0.052*	
N4	0.9663 (4)	0.6910 (3)	0.7114 (4)	0.0414 (10)	
H4AN	1.0105	0.6748	0.6607	0.050*	
H4BN	1.0203	0.7291	0.7565	0.050*	
C1	0.9611 (7)	0.6231 (4)	0.7887 (5)	0.0523 (14)	
H1A	0.9198	0.6405	0.8476	0.063*	
H1B	1.0569	0.6051	0.8291	0.063*	
C2	0.8750 (8)	0.5550 (4)	0.7202 (7)	0.0643 (18)	
H2A	0.8905	0.5089	0.7711	0.077*	
H2B	0.9095	0.5423	0.6554	0.077*	
C3	0.7171 (8)	0.5710 (4)	0.6718 (6)	0.0639 (18)	
H3A	0.6679	0.5219	0.6425	0.077*	
H3B	0.6840	0.5898	0.7348	0.077*	
C4	0.5235 (7)	0.6431 (5)	0.5235 (6)	0.0643 (19)	
H4A	0.4797	0.5967	0.4788	0.077*	
H4B	0.4827	0.6510	0.5860	0.077*	
C5	0.4968 (6)	0.7142 (5)	0.4451 (5)	0.065 (2)	
H5A	0.5311	0.7049	0.3791	0.078*	
H5B	0.3959	0.7252	0.4147	0.078*	
C6	0.5603 (7)	0.8554 (5)	0.4447 (5)	0.0629 (19)	
H6A	0.4607	0.8690	0.4110	0.076*	
H6B	0.5972	0.8452	0.3805	0.076*	
C7	0.6384 (8)	0.9245 (4)	0.5152 (7)	0.068 (2)	
H7A	0.6117	0.9719	0.4677	0.082*	
H7B	0.6070	0.9311	0.5833	0.082*	
C8	0.7971 (7)	0.9183 (4)	0.5569 (6)	0.0586 (16)	
H8A	0.8289	0.9063	0.4904	0.070*	
H8B	0.8372	0.9690	0.5885	0.070*	
O1	0.2360 (12)	0.5429 (5)	0.5295 (7)	0.143 (3)	
O2	0.1991 (14)	0.4382 (5)	0.6357 (8)	0.183 (5)	
O3	0.3986 (11)	0.5079 (10)	0.6805 (16)	0.257 (8)	
O4	0.2177 (16)	0.5591 (8)	0.7161 (12)	0.231 (7)	
O5	1.1848 (9)	0.8171 (7)	0.7049 (9)	0.173 (5)	
O6	1.1523 (12)	0.9046 (7)	0.5676 (8)	0.186 (5)	
O7	1.2769 (14)	0.7974 (8)	0.5576 (17)	0.277 (10)	
O8	1.3760 (9)	0.8831 (8)	0.6872 (10)	0.188 (5)	
O1W	0.8322 (4)	0.7379 (3)	0.4706 (3)	0.0412 (9)	
H1OA	0.782 (7)	0.743 (4)	0.398 (6)	0.07 (2)*	
H1OB	0.896 (6)	0.714 (4)	0.474 (5)	0.046 (19)*	
O2W	1.0660 (6)	0.6849 (4)	0.4419 (5)	0.0841 (17)	

### Atomic displacement parameters (Å^2^)

**Table:**

	*U*^11^	*U*^22^	*U*^33^	*U*^12^	*U*^13^	*U*^23^
Cr	0.0281 (4)	0.0384 (4)	0.0229 (4)	−0.0002 (3)	0.0098 (3)	−0.0013 (3)
Cl1	0.0632 (9)	0.0531 (8)	0.0533 (8)	−0.0025 (7)	0.0302 (7)	−0.0047 (6)
Cl2	0.0436 (8)	0.0758 (11)	0.0728 (11)	−0.0005 (7)	0.0175 (7)	0.0124 (9)
F	0.0427 (16)	0.0559 (18)	0.0266 (13)	−0.0032 (13)	0.0166 (12)	−0.0043 (12)
N1	0.049 (3)	0.050 (3)	0.044 (2)	−0.015 (2)	0.022 (2)	−0.013 (2)
N2	0.032 (2)	0.073 (3)	0.032 (2)	0.010 (2)	0.0118 (17)	0.001 (2)
N3	0.048 (3)	0.040 (2)	0.048 (3)	−0.0004 (19)	0.022 (2)	−0.0008 (19)
N4	0.035 (2)	0.051 (3)	0.037 (2)	0.0042 (19)	0.0107 (18)	0.0039 (19)
C1	0.064 (4)	0.053 (3)	0.042 (3)	0.014 (3)	0.019 (3)	0.010 (3)
C2	0.083 (5)	0.036 (3)	0.078 (5)	0.007 (3)	0.032 (4)	0.011 (3)
C3	0.082 (5)	0.048 (3)	0.071 (4)	−0.019 (3)	0.038 (4)	−0.002 (3)
C4	0.043 (3)	0.082 (5)	0.068 (4)	−0.026 (3)	0.018 (3)	−0.027 (4)
C5	0.033 (3)	0.113 (6)	0.041 (3)	−0.008 (3)	0.001 (2)	−0.018 (4)
C6	0.048 (3)	0.097 (5)	0.045 (3)	0.030 (3)	0.017 (3)	0.029 (3)
C7	0.077 (5)	0.060 (4)	0.077 (5)	0.033 (4)	0.036 (4)	0.022 (4)
C8	0.067 (4)	0.044 (3)	0.077 (4)	0.008 (3)	0.041 (4)	0.012 (3)
O1	0.220 (10)	0.118 (6)	0.095 (5)	−0.040 (6)	0.058 (6)	−0.005 (5)
O2	0.337 (15)	0.099 (6)	0.131 (7)	−0.105 (8)	0.099 (9)	−0.011 (5)
O3	0.109 (8)	0.285 (16)	0.40 (2)	0.041 (10)	0.120 (11)	0.135 (16)
O4	0.320 (17)	0.204 (12)	0.246 (13)	−0.030 (11)	0.198 (13)	−0.117 (10)
O5	0.113 (6)	0.241 (12)	0.196 (10)	−0.005 (7)	0.091 (7)	0.089 (9)
O6	0.198 (10)	0.226 (12)	0.105 (6)	0.110 (9)	0.007 (6)	0.029 (7)
O7	0.226 (13)	0.185 (11)	0.53 (3)	−0.062 (9)	0.278 (17)	−0.190 (15)
O8	0.091 (6)	0.270 (13)	0.173 (9)	−0.082 (7)	0.002 (5)	0.040 (9)
O1W	0.0369 (19)	0.063 (2)	0.0266 (17)	0.0085 (18)	0.0144 (15)	0.0048 (16)
O2W	0.061 (3)	0.110 (5)	0.088 (4)	0.030 (3)	0.033 (3)	0.003 (3)

### Geometric parameters (Å, °)

**Table:**

Cr—F	1.881 (3)	N4—H4BN	0.900
Cr—O1W	1.997 (3)	C1—C2	1.511 (10)
Cr—N1	2.068 (5)	C1—H1A	0.970
Cr—N2	2.070 (4)	C1—H1B	0.970
Cr—N3	2.082 (5)	C2—C3	1.516 (10)
Cr—N4	2.095 (4)	C2—H2A	0.970
Cl1—O4	1.282 (8)	C2—H2B	0.970
Cl1—O3	1.377 (11)	C3—H3A	0.970
Cl1—O2	1.392 (8)	C3—H3B	0.970
Cl1—O1	1.405 (8)	C4—C5	1.498 (11)
Cl2—O7	1.321 (10)	C4—H4A	0.970
Cl2—O8	1.364 (8)	C4—H4B	0.970
Cl2—O5	1.365 (7)	C5—H5A	0.970
Cl2—O6	1.381 (9)	C5—H5B	0.970
N1—C3	1.496 (8)	C6—C7	1.504 (11)
N1—C4	1.502 (8)	C6—H6A	0.970
N1—H1AN	0.910	C6—H6B	0.970
N2—C6	1.484 (8)	C7—C8	1.499 (10)
N2—C5	1.488 (8)	C7—H7A	0.970
N2—H1N2	0.910	C7—H7B	0.970
N3—C8	1.488 (8)	C8—H8A	0.970
N3—H3AN	0.900	C8—H8B	0.970
N3—H3BN	0.900	O1W—H1OA	0.86 (6)
N4—C1	1.487 (7)	O1W—H1OB	0.74 (6)
N4—H4AN	0.900		
			
F—Cr—O1W	179.49 (16)	N4—C1—C2	112.0 (5)
F—Cr—N1	89.72 (16)	N4—C1—H1A	109.2
O1W—Cr—N1	90.30 (18)	C2—C1—H1A	109.2
F—Cr—N2	88.63 (15)	N4—C1—H1B	109.2
O1W—Cr—N2	90.87 (17)	C2—C1—H1B	109.2
N1—Cr—N2	84.3 (2)	H1A—C1—H1B	107.9
F—Cr—N3	89.77 (16)	C1—C2—C3	114.3 (5)
O1W—Cr—N3	90.17 (18)	C1—C2—H2A	108.7
N1—Cr—N3	176.23 (19)	C3—C2—H2A	108.7
N2—Cr—N3	91.9 (2)	C1—C2—H2B	108.7
F—Cr—N4	91.04 (15)	C3—C2—H2B	108.7
O1W—Cr—N4	89.47 (17)	H2A—C2—H2B	107.6
N1—Cr—N4	90.98 (19)	N1—C3—C2	112.4 (5)
N2—Cr—N4	175.3 (2)	N1—C3—H3A	109.1
N3—Cr—N4	92.77 (19)	C2—C3—H3A	109.1
O4—Cl1—O3	108.6 (11)	N1—C3—H3B	109.1
O4—Cl1—O2	113.8 (8)	C2—C3—H3B	109.1
O3—Cl1—O2	106.6 (9)	H3A—C3—H3B	107.9
O4—Cl1—O1	119.5 (9)	C5—C4—N1	108.7 (5)
O3—Cl1—O1	97.5 (8)	C5—C4—H4A	109.9
O2—Cl1—O1	108.9 (5)	N1—C4—H4A	109.9
O7—Cl2—O8	104.5 (8)	C5—C4—H4B	109.9
O7—Cl2—O5	114.7 (9)	N1—C4—H4B	109.9
O8—Cl2—O5	112.8 (7)	H4A—C4—H4B	108.3
O7—Cl2—O6	110.5 (10)	N2—C5—C4	107.8 (5)
O8—Cl2—O6	110.7 (8)	N2—C5—H5A	110.1
O5—Cl2—O6	103.7 (7)	C4—C5—H5A	110.1
C3—N1—C4	111.9 (5)	N2—C5—H5B	110.1
C3—N1—Cr	117.1 (4)	C4—C5—H5B	110.1
C4—N1—Cr	107.0 (4)	H5A—C5—H5B	108.5
C3—N1—H1AN	106.8	N2—C6—C7	112.8 (5)
C4—N1—H1AN	106.8	N2—C6—H6A	109.0
Cr—N1—H1AN	106.8	C7—C6—H6A	109.0
C6—N2—C5	112.3 (5)	N2—C6—H6B	109.0
C6—N2—Cr	119.7 (4)	C7—C6—H6B	109.0
C5—N2—Cr	106.8 (4)	H6A—C6—H6B	107.8
C6—N2—H1N2	105.7	C8—C7—C6	115.7 (6)
C5—N2—H1N2	105.7	C8—C7—H7A	108.4
Cr—N2—H1N2	105.7	C6—C7—H7A	108.4
C8—N3—Cr	119.0 (4)	C8—C7—H7B	108.4
C8—N3—H3AN	107.6	C6—C7—H7B	108.4
Cr—N3—H3AN	107.6	H7A—C7—H7B	107.4
C8—N3—H3BN	107.6	N3—C8—C7	112.7 (5)
Cr—N3—H3BN	107.6	N3—C8—H8A	109.0
H3AN—N3—H3BN	107.0	C7—C8—H8A	109.0
C1—N4—Cr	116.9 (4)	N3—C8—H8B	109.0
C1—N4—H4AN	108.1	C7—C8—H8B	109.0
Cr—N4—H4AN	108.1	H8A—C8—H8B	107.8
C1—N4—H4BN	108.1	Cr—O1W—H1OA	129 (5)
Cr—N4—H4BN	108.1	Cr—O1W—H1OB	117 (5)
H4AN—N4—H4BN	107.3	H1OA—O1W—H1OB	109 (6)

### Hydrogen-bond geometry (Å, °)

**Table:**

*D*—H···*A*	*D*—H	H···*A*	*D*···*A*	*D*—H···*A*
O1W—H1OA···F^i^	0.86 (6)	1.72 (6)	2.564 (5)	168 (8)
O1W—H1OB···O2W	0.74 (6)	1.92 (6)	2.617 (6)	158 (7)
N1—H1AN···O2^ii^	0.91	2.42	3.332 (11)	177
N2—H1N2···O7^iii^	0.91	2.45	3.154 (10)	134
N3—H3BN···O5	0.90	2.36	3.242 (10)	167
N4—H4BN···O5	0.90	2.43	3.062 (10)	127
C1—H1B···O4^iv^	0.97	2.53	3.141 (13)	121

Symmetry codes: (i) *x*, −*y*+3/2, *z*−1/2; (ii) −*x*+1, −*y*+1, −*z*+1; (iii) *x*−1, *y*, *z*; (iv) *x*+1, *y*, *z*.
